# Effect of Different Additives on Genotoxicity of Mineral Trioxide Aggregate 

**DOI:** 10.22037/iej.v13i1.16913

**Published:** 2018

**Authors:** Mohammad Samiei, Shahriar Shahi, Negin Ghasemi, Siavoush Dastmalchi, Nasrin Bargahi, Saeed Asgary

**Affiliations:** a *Department of Endodontics, Dental School, Tabriz University of Medical Sciences, Tabriz, Iran; *; b *Department of Endodontics, Dental and Periodontal Research Center, Dental School, Tabriz University of Medical Sciences, Tabriz, Iran; *; c *Department of Medicinal Chemistry, School of Pharmacy, Tabriz University of Medical Sciences, Tabriz, Iran; *; d *Department of Genetic, Biotechnology Research Center, Tabriz University of Medical Sciences, Tabriz, Iran;*; e * Iranian Center for Endodontic Research, Research Institute of Dental Sciences, Dental School, Shahid Beheshti University of Medical Sciences, Tehran, Iran*

**Keywords:** Ames Test, Disodium Hydrogen Phosphate, Genotoxicity, Mineral Trioxide Aggregate, Nano Silver

## Abstract

**Introduction:**

The aim of the present *in vitro* study was to evaluate the genotoxicity of mineral trioxide aggregate (MTA) after adding different concentrations of disodium hydrogen phosphate and silver nanoparticles using the Ames test.

**Methods and Materials:**

TA100 strain of *Salmonella typhimurium *was used to evaluate mutagenicity of experimental materials with and without S9 mix fraction. The materials tested in this study consisted of MTA, MTA/disodium hydrogen phosphate and MTA/silver nanoparticles at 0.1, 0.01, 0.001 and 0.0001 concentrations. Negative and positive control groups consisted of 1% dimethyl sulfoxide and sodium azide with 2-aminoanthracene, respectively. The number of colonies per plate was determined. If the ratio of the number of histidine-revertant colonies to spontaneous revertants of the negative control colonies was ≥2, the material was regarded a mutagenic agent.

**Results:**

In all the concentrations of the three tested materials, the Ames test failed to detect mutations.

**Conclusion:**

Under the limitations of the present study, MTA/disodium hydrogen phosphate and MTA/silver nanoparticles were biocompatible in relation to mutagenicity.

## Introduction

Mineral Trioxide Aggregate (MTA) is a calcium silicate-based biomaterial and is widely used in endodontics due to its favorable properties, including sealing ability [1], setting capacity in the presence of blood/moisture [2] bio-compatibility [3, 4], and the ability to induce regeneration in the pulp and periapical tissues [5]. Its applications in endodontics include as a pulp covering agent, as an apical plug/root-end fillings and as a perforation repair biomaterial. However, MTA has a long setting time and its handling is difficult [6]. Some studies have shown its poor antimicrobial properties against resistant bacteria such as *Enterococcus faecalis* [7].

Several studies have evaluated and suggested different strategies to improve the properties of MTA. One strategy is to incorporate ingredients into MTA to decrease its setting time. Disodium hydrogen phosphate is a buffering agent which has an effect on decreasing the setting time of MTA [8, 9].

Due to the antibacterial properties of silver nanoparticles, they have been incorporated into MTA [10]. These particles have a high surface-to-volume ratio and when added in small amounts interfere with peptidoglycans of bacterial cell wall and increase their permeability. In addition, they react with the sulfhydryl groups of bacterial proteins and prevent DNA replication, resulting in bacterial death [11]. 

When ingredients are incorporated into a biomaterial to improve its properties, important factors that should be taken into account are the cytotoxicity and genotoxicity of the final product [12]. Studies on the cytotoxicity of MTA after incorporation of disodium hydrogen phosphate have shown its biocompatibility [13-15]. In addition, incorporation of silver nanoparticles at 1 wt% and at 23-47 ppm concentrations has resulted in tissue reactions similar to those mounted to MTA alone; *i.e.* the resultant material has been shown to be biocompatible [10, 16]. Also, due to the results reported by Li *et al. *[17], the Ames was negative regarding the genotoxicity of silver nanoparticles and suggested that this result can be due to the inability of these nanoparticles to penetrate the bacterial cell wall.

No study is available on the genotoxicity of MTA after incorporation of disodium hydrogen phosphate and silver nanoparticles. Genotoxicity is defined as the induction of gene damage, gene mutation and chromosomal breakage. Of all the tests that evaluate genotoxicity, those that evaluate gene mutations are very reliable. The *Salmonella typhimurium *assay or Ames test is a bacterial test widely used to determine the potency of substances that can produce genetic damage and gene mutations [18, 19]. The Ames test utilizes *Salmonella *strains with preexisting mutations that are unable to synthesize the required amino acid, histidine, and therefore cannot grow and form colonies in the medium devoid of histidine. New mutations at the site of these preexisting mutations can restore the genes’ function and allow the cells to synthesize histidine. These newly mutated cells can grow in the absence of histidine and form colonies. The *Salmonella *mutagenicity test was specifically designed to detect chemically induced mutagenesis [20, 21].

The aim of the present *in vitro *study was to evaluate the genotoxicity of MTA, MTA/disodium hydrogen phosphate and MTA/silver nanoparticles using Ames test.

## Materials and Methods

In this study, TA100 strain of *Salmonella typhimurium *was used in order to investigate mutagenicity of MTA (Angelus Dental Industry Products, Londrina, Brazil), MTA/silver nanoparticles (produced in the laboratory)[21], and MTA/disodium hydrogen phosphate (Merck, Darmstad, Germany) with and without S9 mix fraction.

MTA, MTA/silver nanoparticles (average nanoparticle size of 20 nm) and MTA/disodium hydrogen phosphate were prepared at 0.1, 0.01, 0.001 and 0.0001 concentrations [21].

The *Salmonella typhimurium *bacterial strain used in the Ames test carried a mutant gene preventing it from synthesizing the essential amino acid histidine from the ingredients found in the standard bacterial culture medium. In this study, negative and positive control groups were included to assess the accuracy of the tests. The negative control group consisted of 1% dimethyl sulfoxide (DMSO) and distilled water. The positive control group, used to compare the results, consisted of sodium azide (0.5 µg/plate) and 2-aminoanthracene (2.5 µg/plate) as strong mutagenic agents in the absence of metabolic and metabolic activation systems. The TA100 strain of *Salmonella typhimurium* was cultured overnight in nutrient broth. Different concentrations of the test materials were prepared in a histidine-free medium. Glucose minimal (GM) agar plate consisted of distilled water, agar, Vogel-Bonner (VB) salt solution and glucose solution (10% v/v). VB salt medium consisted of warm distilled water, magnesium sulfate, citric acid monohydrate, potassium phosphate dibasic anhydrous, and sodium ammonium phosphate. Before the incubation, each test material and *Salmonella typhimurium *(50 µl) were incorporated into 2 mL of top agar containing distilled water, agar, sodium chloride and histidine/biotin solution (0.5 mM). The mixture was poured on GM agar plates. After solidification of the superficial agar, the plates were incubated at 37^°^C for 48 to 72 h. After proliferation of bacteria on the glucose-minimal agar plate, the histidine-revertant clones were counted using a colony counter. Rat liver enzymes (S9) were used as a metabolic activator. The microsomal enzyme system was induced by phenobarbital in rats and the rat liver was homogenized after 7 days in order to isolate the enzyme. A suspension of *Salmonella typhimurium* with a mixture of rat liver enzymes (S9 mix) and different cofactors, such as glucose-6 phosphate and NADP, were added to the superficial agar. Subsequently, the plates were incubated for 48 to 72 h in 37^°^C without shaking. Then the number of colonies was counted per plate. The ratio of the number of histidine-revertant colonies to spontaneous revertants of the negative control colonies was obtained; if the ratio was ≥2, the experimental material was considered as a mutagenic agent.

## Results

Table 1 presents the number of initial and secondary colonies and their ratios (S9+/S9-). In all experimental groups and at all the concentrations these ratios were <2; therefore, there was no genotoxicity and there was no need for statistical analysis. The findings of positive and negative controls were as expected in the present study. 

## Discussion

In this *in vitro* study, the genotoxicity of different concentrations of MTA/silver nanoparticles and MTA/disodium hydrogen phosphate was evaluated, using Ames test. The results showed the safety of these materials in relation to genotoxicity. MTA is a silicate-based cement with numerous therapeutic applications in endodontics. Efforts have always been made to achieve favorable clinical outcomes by improving the physicochemical properties of MTA [22, 23]. Some studies have been undertaken in an attempt to decrease its setting time and increase its antimicrobial activity, which are prerequisites for achieving favorable treatment outcomes. In this context, some materials have been incorporated into the MTA powder.

One of the materials incorporated into the MTA composition was disodium hydrogen phosphate. Based on the results of previous studies, incorporation of this material into MTA results in a decrease in setting time, in an increase in tensile strength and in an increase in push-out bond strength; however, the setting pH is not affected [8, 9, 13, 24]. Studies on the cytotoxicity of this composition have reported it to be biocompatible [14, 15, 25]. Previous studies have used 2.5 and 15 wt% of disodium hydrogen phosphate [8, 26], which is different from the present study (100-200 ppm and %1 weight) and it has been recommended that future studies on the genotoxicity of MTA be carried out by incorporating these weight percentages of disodium hydrogen phosphate.

Silver ions have drawn attention in the medical field as significant antimicrobial agents. Silver nanoparticles exhibit less toxicity compared to silver ions due to their small size and high surface-to-volume ratio at a similar concentration, with antimicrobial activity comparable to that of silver ions [10, 16]. Studies have shown that incorporation of silver nanoparticles 

(100-200 ppm and %1 weight) into MTA results in an increase in their antibacterial activity against *E. faecalis* and *C. albicans* [11, 27], which are microorganisms resistant to antimicrobial agents and are found in the majority of cases of endodontic failures [28-30]. Silver is a toxic agent and its toxicity is concentration-dependent [23]. Previous studies have shown that concentrations of 23-47 ppm are safe. In addition, incorporation of silver nanoparticles into MTA at 1 wt% did not result in a significant difference in the inflammatory reactions induced in rat connective tissue compared to pure MTA [15]. In the present study, incorporation of silver nanoparticles at weight percentages mentioned above did not result in genotoxicity.

**Table 1:** Mean revertant colony counts after exposure to four concentrations of test materials with and without S9 mix fraction

**Experimental materials**			**Colony counts of TA100**		**Ratio of TA 100**		
			**S9+**	**S9-**	**S9+**	**S9-**
**MTA**	**0.1**	250		335	0.8	0.7
	**0.01**	58		11	0.1	0.6
	**0.001**	85		200	0.2	0.5
	**0.0001**	60		250	0.1	1.45
**MTA/NaHPO**_4_	**0.1**	53		0	0.18	0.7
	**0.01**	83		200	0.2	0.6
	**0.001**	350		650	1	0.5
	**0.0001**	395		200	1.27	1.45
**MTA/Silver nanoparticles**	**0.1**	400		405	1.3	0.8
	**0.01**	3		43	0.2	0.07
	**0.001**	80		660	0.2	1.8
	**0.0001**	NA		NA	NA	NA

In many studies, the biocompatibility of silver nanoparticles and disodium hydrogen phosphate was evaluated by placing their implants in the subcutaneous tissues of rats or in exposure with cells in the cultures media; the histological cross-sections showed the safety of these materials [12-15, 25]. However, the genotoxicity and mutation induction capacity of these materials have not been evaluated to date. Ames test was used in the present study to evaluate the genotoxicity of these materials. In the design of this test, changes are induced in the genome area of *Salmonella* responsible for production of histidine. Therefore, these mutagenic strains cannot proliferate in an environment without histidine; however, if a material has mutagenic activity, it can result in the repair of the iatrogenic defect in the bacterial genome and the bacteria will be able to synthesize histidine again and will be able to proliferate in the culture medium. If the ratio of these new colonies to the initial colonies is ≥2, the material is considered to be mutagenic [20, 21]. In the present study, this test was used and none of the materials was shown to be mutagenic. 

## Conclusion

Based on the results of the present *in vitro* study, MTA/silver nanoparticles and MTA/disodium hydrogen phosphate had no genotoxicity at the concentrations used.
